# Full genome sequence of a human rhinovirus A1B, obtained in Kazakhstan

**DOI:** 10.1128/MRA.00749-23

**Published:** 2023-10-05

**Authors:** Vitaliy Strochkov, Vyacheslav Beloussov, Maxim Solomadin, Joanna Granica, Sergey Yegorov, Shynggys Orkara, Nurlan Sandybayev

**Affiliations:** 1 Kazakhstan-Japan Innovation Centre, Kazakh National Agrarian Research University (KazNARU), Almaty, Kazakhstan; 2 TreeGene Molecular Genetics Laboratory, Almaty, Kazakhstan; 3 School of Pharmacy, Karaganda Medical University, Karaganda, Kazakhstan; 4 Department of Biochemistry and Biomedical Sciences, Michael G. DeGroote Institute for Infectious Disease Research, McMaster Immunology Research Centre, McMaster University, Hamilton, Ontario, Canada; 5 School of Sciences and Humanities, Nazarbayev University, Astana, Kazakhstan; DOE Joint Genome Institute, Berkeley, California, USA

**Keywords:** rhinovirus, metagenomics, DNA sequencing

## Abstract

Here, we report the full nucleotide sequence of the RvA1B/KZ/2021/87 rhinovirus, identified through metagenomic sequencing of nasopharyngeal swabs collected from patients exhibiting respiratory symptoms in Kazakhstan during 2021.

## ANNOUNCEMENT

Human rhinovirus (HRV) is a prevalent respiratory pathogen known to cause mild to moderate respiratory illnesses in humans. Its initial identification in 1956 involved patients with mild upper respiratory tract infections (1), and it has since emerged as a primary causative agent of the common cold across different age groups. Belonging to the Picornaviridae family within the *Enterovirus* genus, HRV encompasses three distinct species: HRV-A, HRV-B, and HRV-C (2 – 5). This viral diversity comprises more than 100 recognized HRV serotypes categorized within these species, characterized by nucleotide sequence variations that potentially contribute to the varied clinical manifestations of HRV infections (6).

Nasopharyngeal swabs were collected in the Almaty region during May–June 2021 from patients displaying respiratory infection symptoms as part of an ongoing project aimed at elucidating the upper respiratory tract virome in respiratory infections (7). All study procedures were approved by the Commission on Bioethics of KazNARU (dated 15 October 2020). Written informed consent was obtained from all participants.

The extracted nucleic acids from the collected samples were processed using the MagMAX Total Nucleic Acid Isolation Kit (Thermo Fisher Scientific, Waltham, MA, USA). Subsequently, cDNA synthesis, amplification, and primer removal were performed using the SeqPlex RNA Amplification Kit (Sigma, St. Louis, MO, USA) to generate cDNA fragments ranging from 150 to 400 nucleotides in length. Quantity and quality of the amplified cDNA products were evaluated through NanoDrop and Qubit assessments. Libraries suitable for downstream analysis were prepared using the Ion Plus Fragment Library Kit (Thermo Fisher Scientific).

**Fig 1:**
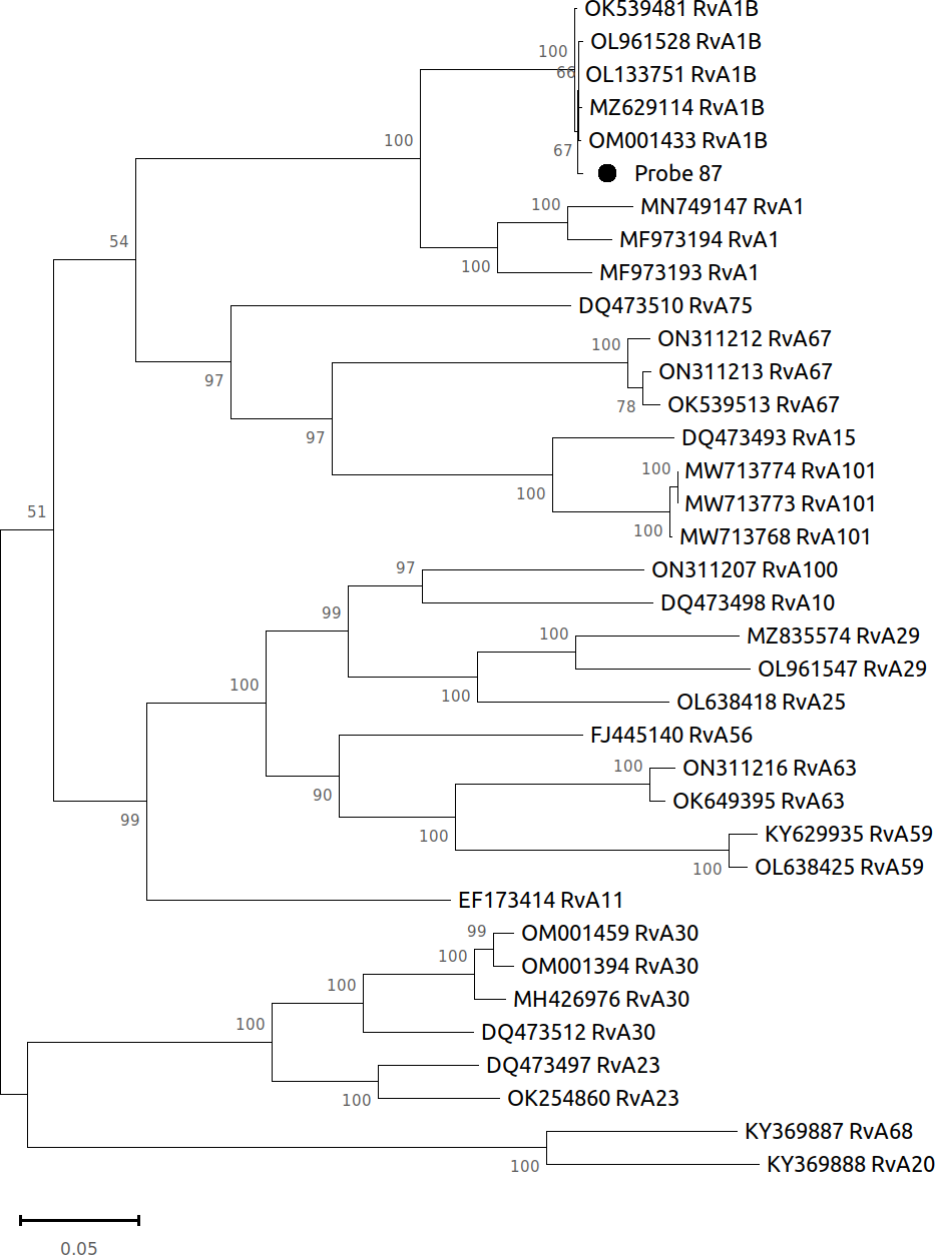
Phylogenetic tree complete nucleotide sequence of RvA1B/KZ/2021/87. Evolutionary insights were derived through the maximum likelihood method, using the Tamura-Nei model (10). The tree is drawn to scale, with branch lengths measured in the number of substitutions per site. This analysis involved 36 nucleotide sequences of complete genomes.

Sequencing was executed using the Ion S5 platform, a next-generation sequencing technology. Quality control, trimming, removal of human DNA, and taxonomic classification of the resulting sequence were performed using CZ ID NGS Pipeline v.7.1 (formerly IDseq, available at https://czid.org) (8), facilitating precise categorization. A total of 147,931 high-quality reads with an average length of 200 bp were obtained, corresponding to the rhinovirus. To achieve the complete nucleotide sequence of RvA1B/KZ/2021/87, a reference-based assembly approach was performed using the Sequencher v.5.4.6 program (Gene Codes Corporation, Ann Arbor, MI, USA). As a reference, we utilized the RvA1B/USA/2021/PSC7PE strain (GenBank ID: OL133751). The assembled genome of RvA1B/KZ/2021/87 encompasses a length of 7,047 base pairs. Notably, the genome displayed a GC content of 38.21% while achieving full genome coverage at a depth exceeding 1,000×. Upon employing the BLAST program for sequence comparison, it was revealed that the nucleotide sequences of rhinoviruses isolated in the USA during 2021 bear the closest resemblance. Specifically, the highest degree of nucleotide identity, at 99.69%, was observed with the RvA1B/USA/2021/PSC7PE strain (GenBank ID: OL133751). All tools were run with default parameters unless otherwise specified. The analytical framework for evolutionary assessments was conducted within the MEGA11 software (9). Visual representations of the inferred phylogenetic relationships are depicted in Fig. 1.

## Data Availability

The nucleotide sequence is available under the accession number OP886969 at the National Center for Biotechnology Information (NCBI) database. The raw sequencing reads have been submitted to the NCBI's Sequence Read Archive under the accession number SRR22402249.
